# The effect of exercise during pregnancy on gestational diabetes mellitus in normal-weight women: a systematic review and meta-analysis

**DOI:** 10.1186/s12884-018-2068-7

**Published:** 2018-11-12

**Authors:** Wai-Kit Ming, Wenjing Ding, Casper J. P. Zhang, Lieqiang Zhong, Yuhang Long, Zhuyu Li, Cong Sun, Yanxin Wu, Hanqing Chen, Haitian Chen, Zilian Wang

**Affiliations:** 1 Department of Obstetrics and Gynaecology, The First Affiliated Hospital of Sun Yat-sen University, Guangzhou, China; 2 Harvard Medical School, Harvard University, Boston, MA USA; 3 Division of Pharmacoepidemiology and Pharmacoeconomics, Brigham and Women’s Hospital, Boston, MA USA; 4 School of Public Health, The University of Hong Kong, Hong Kong, China

**Keywords:** Exercise, Gestational diabetes mellitus, Systematic review, Meta-analysis

## Abstract

**Background:**

Gestational diabetes mellitus (GDM) is one of the most common complications during pregnancy, and it has both short- and long-term adverse effects on the health of mothers and fetuses. To investigate the effect of exercise during pregnancy on the occurrence of GDM among normal-weight pregnant women.

**Methods:**

We searched for studies published between January 1994 and June 2017 that appeared in the Web of Science, Scopus, ClinicalTrials.gov or Cochrane library databases. Randomized controlled trials that investigated the preventive effect of exercise on GDM in normal-weight women were included. Interventions including any confounding factors (e.g., dietary) were excluded. We extracted maternal characteristics, the diagnostic criteria of GDM, and basic information for intervention and obstetric outcomes. The primary outcome was the occurrence of GDM, and the secondary outcomes included gestational weight gain, gestational age at birth, birth weight, and the odds of cesarean section. A meta-analysis was conducted based on calculations of pooled estimates using the random-effects model.

**Results:**

Eight studies were included in this systematic review and meta-analysis. Exercise during pregnancy was shown to decrease the occurrence of GDM [RR = 0.58, 95% CI (0.37, 0.90), *P* = 0.01 and RR = 0.60, 95% CI (0.36, 0.98), *P* = 0.04 based on different diagnosis criteria, respectively] in normal-weight women. Regarding secondary outcomes, exercise during pregnancy can decrease gestational weight gain [MD = − 1.61, 95% CI (− 1.99, − 1.22), *P*<0.01], and  had no significant effects on gestational age at birth [MD = − 0.55, 95% CI (− 1.57, 0.47), *P* = 0.29], birth weight [MD = − 18.70, 95% CI (− 52.49, 15.08), *P* = 0.28], and the odds of caesarean section [RR = 0.88, 95% CI (0.72, 1.08), *P* = 0.21], respectively.

**Conclusions:**

Exercise during pregnancy can ostensibly decrease the occurrence of GDM without reducing gestational age at delivery and increasing the odds of cesarean section in normal-weight women.

**Electronic supplementary material:**

The online version of this article (10.1186/s12884-018-2068-7) contains supplementary material, which is available to authorized users.

## Background

Gestational diabetes mellitus (GDM) is a common complication of pregnancy; based on the diagnosis criteria published by the International Association of Diabetes and Pregnancy Study Groups (IADPSG), the estimated prevalence of GDM worldwide is 17.8% [1]. In 2013, the World Health Organization (WHO) adopted the IADPSG evidence-based criteria as their standard for GDM diagnosis [2, 3]; these criteria use lower thresholds for several indices (i.e., a fasting glucose ≥5.1 mmol/l, or a one-hour result ≥10.0 mmol/l, or a two-hour result ≥8.5 mmol/l, using a 75 g oral glucose tolerance test) than previously accepted, therefore yielding more cases of GDM [4]. Gestational diabetes mellitus is more common among women who are overweight or of advanced maternal age, have a history of GDM and macrosomia, and who have a family history of diabetes [5–8].

Gestational diabetes mellitus can affect the health of mothers and their offspring due to transient abnormalities in carbohydrate metabolism [1, 5, 9, 10]. Women with GDM are at higher risk of experiencing fetal demise, fetal malformation, preterm birth, macrosomia, polyhydramnios, infection, and cesarean section than the general population [11–15]. Furthermore, both women with GDM and their infants are more likely to become overweight or obese [16, 17] and develop type 2 diabetes mellitus (T2DM) [10], cardiovascular diseases (CVD) and neuropsychological deficits later in life than the normal group [1, 17, 18].

Recent studies have demonstrated that GDM could be modified by lifestyle interventions such as exercise and diet control [19]. Exercise is characterized as planned, structured, repetitive movement that has a specific goal (e.g., health improvement). It is a subcategory of physical activity, which refers to any movement that involves energy expenditures and the use of skeletal muscles [20]. Exercise is deemed to be an important component of lifestyle intervention for GDM [21]. Regular exercise reduces the risk of T2DM, CVD, and metabolic syndrome in non-pregnant patients [22, 23]. The Royal College of Obstetricians and Gynecologists (RCOG) recommends that to accrue health benefits, healthy pregnant women should engage at least 30 min of moderate-intensity exercise at least four times per week [24]. However, only a small proportion of pregnant women achieve this goal.

Several meta-analyses support the evidence that exercise protects against GDM. Da Silva et al. concluded that leisure-time physical activity during pregnancy played a protective role against the development of GDM [25]; another meta-analysis of randomized controlled trials (RCTs) found that exercise prevents GDM in normal-weight and overweight women [26]; yet another meta-analysis of the association between exercise and preterm birth also showed that exercise lowers the occurrence of GDM in overweight or obese women [27].

### Objectives

The majority of the GDM population comprises women of normal weights (based on pre-pregnant body mass index [BMI]). However, the existing systematic reviews and meta-analyses focused on the over-weight or obese population [28]. Exclusively on the normal-weight population, there are few reviews of pregnancy outcomes and one recent paper focused on the exercise during pregnancy in the normal-weight population and the risk of preterm birth [29]. To our knowledge, no published reviews have examined GDM in such population. Evidence of how exercise influencing GDM in normal-weight women could inform first-line treatments of GDM in clinical practice. Earlier meta-analyses of all-weight populations did not rule out the impact of maternal weight on GDM given that overweight and obese populations are at high risk of GDM and their status may be attributed to a variety of factors. Here, we synthesized available evidence of RCTs of exercise during pregnancy in preventing GDM in normal-weight women.

## Methods

We conducted a systematic review and meta-analysis and reported our findings according to the Preferred Reporting Items for Systematic Reviews and Meta-Analyses (PRISMA) statement.

### Search strategy

We searched Web of Science, Scopus (including Pubmed, MEDLINE and Embase), ClinicalTrials.gov and the Cochrane Library for articles published between January 1994 and June 2017, using the following combinations of keywords: (‘activit*’ OR ‘fitness’ OR ‘exercise’ OR ‘sport*’ OR ‘physical activit*’ OR ‘physical exercise’) AND (‘pregnancy’ OR ‘wom*’) AND (‘trial*’) AND (‘diabetes’ OR ‘gestational diabetes’ OR ‘gestational diabetes mellitus’ OR ‘GDM’ OR ‘glucose’). The integrated search strategy is shown in Additional file 1: Textbox 1. These search terms were reviewed by a trained librarian and a physician. Reference citations for relevant articles were additionally screened to identify possible missing publications.

### Study selection

Studies were included if they satisfied the following conditions: 1) they consisted of randomized controlled trials; 2) interventions used in the study included at least one type of exercise; 3) the occurrence of GDM was reported for both the intervention and control groups; 4) subjects were pregnant women with a pre-pregnancy BMI or a mean pre-pregnancy BMI ranging from 18.5–24.9 kg/m^2^. Publications were excluded if they met any of the following conditions: 1) they integrated interventions of other factors (e.g., dietary) confounding the independent effects of exercise on the occurrence of GDM; 2) the pre-pregnancy BMI or the mean pre-pregnancy BMI of each group was less than 18.5 kg/m^2^ or equal to or greater than 25 kg/m^2^; 3) papers were literature reviews, case reports or protocols; 4) only the abstract or conference contents were published, or the studies lacked specific data.

### Data extraction and outcome measures

Two reviewers (W.D., W.M.) independently searched the literature and extracted data from all eligible studies. Any discrepancy in crosschecks was resolved by a third reviewer and by discussion between all participating authors. The following data were extracted if available: 1) study characteristics (authors, publication year, country, affiliation of the authors, number of subjects, and gestational period); 2) exercise intervention (type, frequency, duration, and intensity); 3) pregnancy outcomes (GDM, gestational weight gain [GWG], caesarean section, gestational age, and birth weight). The primary outcome was the occurrence of GDM, and the secondary outcomes included gestational age at birth, cesarean section, birth weight, and GWG.

### Assessment of risk of Bias

Quality assessment was based on the criteria outlined in the Cochrane Handbook for Systematic Reviews of Interventions and consisted of 1) randomization; 2) concealment of allocation; 3) blinding of the outcome assessment (blinding of participants and healthcare providers was impossible owing to the nature of exercise); 4) incomplete outcome data; 5) selective reporting and 6) other potential bias.

### Data synthesis

Data analysis was conducted using Review Manager 5.3 (RevMan 5.3). Relative risks (RRs) or mean differences (MDs) with 95% confidence intervals (CIs) were used to calculate pooled effects. Relative risks were reported for dichotomous outcomes (i.e., the occurrence of GDM and cesarean section), and MDs were reported for continuous outcomes (i.e., gestational age at birth, gestational weight gain, and birth weight). Heterogeneity was assessed using the Cochran Q statistic (*P* < 0.1), qualified with Higgins I^2^ statistics. A *p*-value less than 0.05 in the two-tailed tests was considered to be statistically significant.

## Results

### Study selection and characteristics

**Fig. 1:**
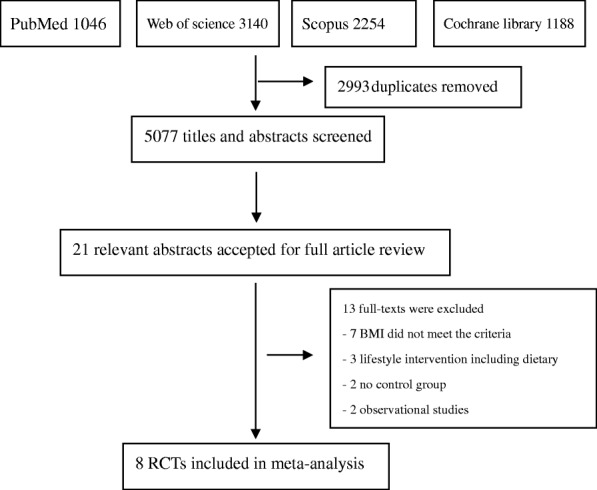
Flow diagram of studies selection

**Fig. 2:**
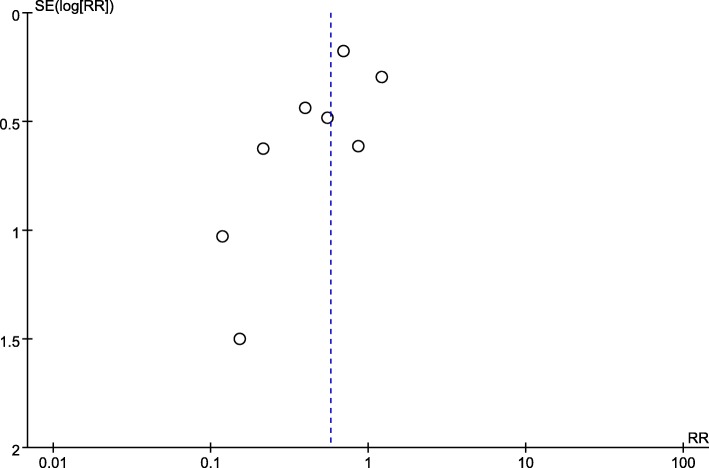
Funnel plot for assessing publication bias SE: standard error; RR: relative risk

We identified 5077 publications in four databases. Upon screening the titles and abstracts, the full texts of 21 studies were reviewed. Of these studies, 13 were excluded due to the following reasons: the pre-pregnancy BMI of the patients did not meet the inclusion criteria (7 studies) [30–36], the patients underwent lifestyle interventions including dietary changes (3 studies) [37–39], there were no control groups (2 studies) [40, 41], or the studies were only observational (2 studies) [42, 43]. Eight RCTs [44–51], including a total of 3256 pregnant women, were eligible for this meta-analysis. The detailed selection procedure is shown in Fig. 1. The publication bias in the primary outcome was assessed by a funnel plot, and the results revealed that such bias existed (Fig. 2).

The general characteristics of the included RCTs are listed in Table 1. All trials were conducted in European countries. The sample sizes ranged from 83 to 962. With the exception of Stafne et al. [50], all of the interventions adopted comprehensive exercise programs of light-to-moderate intensity that were performed three times per week. The duration of each exercise period ranged from 35 to 60 min. Seven trials started in the first trimester and continued to the end of the third trimester [44–49, 51], and only one trial spanned the 20th through 36th weeks of gestation [50]. Pregnant women in the control group received regular antenatal care in all trials.

**Table 1:** Characteristics of randomized controlled trials included in the systematic review and meta-analysis (*n* = 9)

Authors	Year	Country	Subjects(*N*)		Intervention description					Parameters		Diagnosis criteria
			IG	CG	Type of exercise	Gestational period (weeks)	Duration (minutes)	Frequency (times/week)	Intensity	Maternal	Neonatal	
Barakat et al. [45]	2013	Spain	107	93	mobilization exercises, aerobic dance, and muscle training.	from 9 to 13 weeks to the end of the third trimester	50–60	3	Light- moderate	√	√	Not mentioned.
Barakat et al. (a) [51]	2013	Spain	210	218	aerobic exercises, muscle strength and flexibility	weeks 10 to 12 of pregnancy to the end of the third trimester	50–55	3	moderate	√	√	WHO
Barakat et al. (b) [51]	2013	Spain	210	218	aerobic exercises, muscle strength and flexibility	weeks 10 to 12 of pregnancy to the end of the third trimester	50–55	3	moderate	√	√	IADPSG
Barakat et al. [49]	2011	Spain	40	43	two land aerobic sessions and one aquatic activities session.	from week 6–9 to the end of the third trimester	35–45	3	Light- moderate	√	√	Self-reported
Cordero et al. [44]	2014	Spain	101	156	two on land and one as an aquatic activity	weeks 10 and 14 to the end of the third trimester	50–60	3	Light- moderate	√	√	NDDG
Stafne et al. [50]	2012	Norway	375	327	aerobics, resistance, stretching	between 20 and 36 gestation weeks	60	1	Moderate	√	√	WHO
Tomić et al. [48]	2013	Croatia	166	168	aerobic exercise	From 6 to 8 gestation week till the week of delivery	50	3	Moderate	√	√	WHO
Ruiz et al. [47]	2013	Spain	481	481	aerobics exercise and resistance exercises	From week 9 to week 38–39	50–55	3	Light-moderate	√	√	Not mentioned
Barakat et al. [46]	2014	Spain	152	138	toning and joint mobilization exercises and resistance exercises	From week 8–10 to week 38–39	55–60	3	Moderate	√	√	Not mentioned

*IG* intervention group, *CG* control group

Barakat et al. (a) = analysis with World Health Organization criteria; Barakat et al. (b) = analysis with International Association for Diabetes in Pregnancy Study Group criteria

All studies reported the occurrence of GDM, gestational age at birth and birth weight; in addition, gestational weight gain was reported in five studies [45–47, 49, 51]; and the likelihood of caesarean section was reported in seven studies [44, 45, 47–51].

### Risk of Bias in the included studies

**Fig. 3:**
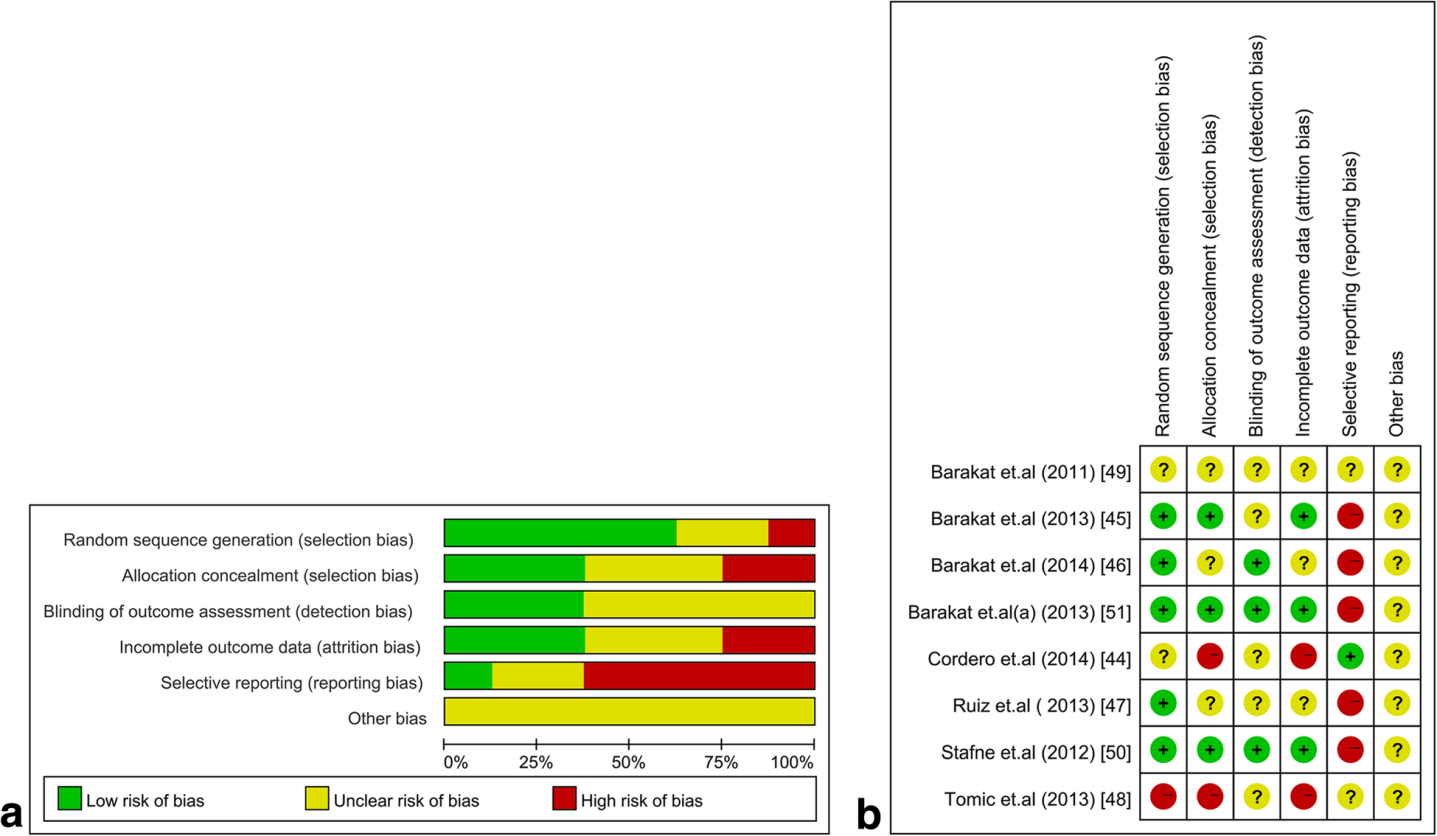
Assessment of risk of bias. (**a**) Risk of bias graph about each risk of bias item presented as percentage across all included studies. (**b**) Summary of risk for bias for each trial; Plus sign: low risk of bias; minus sign: high risk of bias; question mark: unclear risk of bias

Due to the nature of the exercise, blinding of personnel and participants was impractical. We accordingly excluded the blinding component from the bias assessment. Overall, the included trials displayed specific methodological bias (Fig. 3). Five trials showed a low risk of randomization based on the use of a computer random number generator [45–47, 50, 51], and one trial showed a high risk of bias (i.e., randomization is based on the sequence of entering study) [48], and two trials did not report this aspect. Three trials reported that the group allocation was concealed from the staff who conducted the assessment [45, 50, 51]. In terms of incomplete outcome data, three trials reported a full description of participants and follow-up status during the trial [45, 50, 51]. Only one trial was associated with a low risk of selective reporting bias of its outcomes [44].

### Synthesis of results

#### Primary outcome: The occurrence of GDM

The diagnostic criteria for GDM varied among the eight RCTs (among these eight studies, one of the RCT with two criteria, as a result, we decided to did additional analysis as two RCTs): two were based on WHO criteria [50, 51], one was based on IADPSG criteria [51], one was based on National Diabetes Data Group (NDDG) criteria [44], one was based on criteria defined by the authors (self-reported criteria) [49]; and four studies did not report their diagnostic criteria [45–48]. Notably, one trial determined the occurrence of GDM based on the WHO criteria (before 2013) as well as the IADPSG criteria [denoted as “Barakat(a) 2013” and “Barakat(b) 2013,” respectively] (Table 1).

**Fig. 4:**
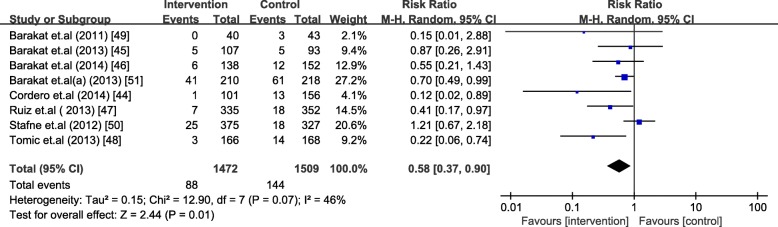
Forest plot for the meta-analysis of the occurrence of gestational diabetes mellitus (1)

**Fig. 5:**
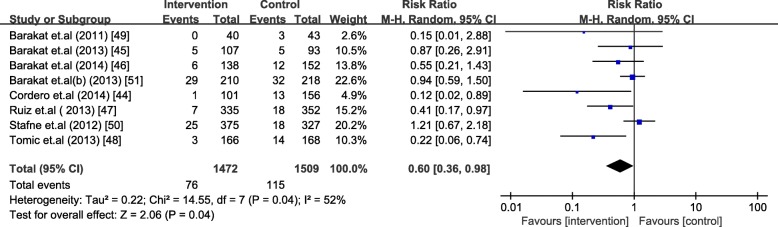
Forest plot for the meta-analysis of the occurrence of gestational diabetes mellitus (2)

The analysis included 1472 women in the intervention group and 1509 women in the control group. Barakat et al. [51] was analyzed as two separate trials due to different diagnosis criteria used. Exercise during pregnancy significantly decreased the occurrence of GDM [RR = 0.58, 95% CI (0.37,0.90), *P* = 0.01 and RR = 0.60, 95% CI (0.36, 0.98), *P* = 0.04 based on different diagnosis criteria, respectively] in normal-weight women. The absolute risk reduction was 3.66% and 2.53%, respectively. The heterogeneity across included studies was high (I^2^ = 46% and 52%, *P* = 0.07 and 0.04) (Figs. 4 and 5).

### Secondary outcomes

Exercise had no significant impact on gestational weight gain [MD = − 1.61, 95% CI (− 1.99, − 1.22), *P*<0.01; Additional file 2: Figure S1], gestational age at birth [MD = − 0.55, 95% CI (− 1.57, 0.47), *P* = 0.29; Additional file 3: Figure S2], birth weight [MD = − 18.70, 95% CI (− 52.49, 15.08), *P* = 0.28; Additional file 4: Figure S3], and the odds of caesarean section [RR = 0.88, 95% CI (0.72, 1.08), *P* = 0.21; Additional file 5: Figure S4].

## Discussion

### Main findings

This meta-analysis of eight studies that included 2981 pregnant women suggests that exercise during pregnancy has a significant protective impact on the occurrence of GDM, and decrease gestational weight gain. Exercise during pregnancy does not reduce the gestational age of delivery or increase the odds of cesarean section in mostly normal-weight pregnant women. The mean gestational age at delivery, birth weight, and the odds of cesarean section are similar in women who exercise regularly and women who receive routine prenatal care. Summary of findings was shown in Additional file 6: Table S1.

### Comparisons with the existing literature

Recently, Shepherd et al. described the effect of combined exercise and diet intervention on preventing GDM in detail, and suggested that combined diet and exercise interventions can reduce risks of GDM [52]. The meta-analysis conducted by Sanabria-Martinez et al., which included all BMI categories, showed that structured exercise during pregnancy could prevent GDM and prevent excessive weight gain [28]. Another meta-analysis performed by Magro-Malosso et al. demonstrated that aerobic exercise during pregnancy, with or without dietary intervention, could reduce the incidence of GDM in overweight and obese women [27]. Recently, Di Mascio et al., in a meta-analysis of nine trials—including 2059 normal-weight women—showed that exercise during pregnancy was not associated with an increased risk of preterm birth. However, these authors found that exercise was correlated with a significantly lower incidence of GDM, cesarean delivery, and hypertensive disorders [29]. All of these studies support our findings. On the other hand, a meta-analysis conducted in 2014 suggested that physical exercise had no significant effect on lowering the occurrence of GDM. However, this study included only 947 pregnant women. In addition, exercise during pregnancy can also decrease the risk of gestational hypertension, preterm birth, cesarean delivery and macrosomia, which can significantly decrease the perinatal morbidity and mortality [53, 54].

### Strengths and limitations

To the best of our knowledge, this study is the first meta-analysis focused on normal-weight women to examine the relationship between exercise/physical activity and the occurrence of GDM. This meta-analysis included all eight RCTs on the topic that have been published so far, with a larger sample size (2981 women) than earlier meta-analyses. Individual studies did not affect the overall results because of the similar sample size of each included study. These key factors are essential for assessing the validity of a meta-analysis.

However, our analysis has some limitations. The baseline characteristics (e.g., maternal age, occupation, educational level, household income, etc.) of the participants across the included studies were not balanced. Furthermore, compliance with the intervention and the effect of exercise might have varied due to differences in maternal education levels, parity, residence, and lifestyle habits before pregnancy; no included study reported adherence and compliance with the exercise regimens. Only one trial was stratified by pre-pregnancy BMI when assessing outcomes [47]; therefore, the mean BMI of the women included in all of the RCTs was in the normal range, but some of the studies might have included a small proportion of underweight, overweight, or obese women. The result of funnel plot suggested possible publication bias, which indicated the effect of exercise during pregnancy on decreasing the risks of GDM was likely reported in published studies, yielding over-estimation of the true effect. Moreover, the method for objectively monitoring physical activity is needed. Cordero et al. [44] used a heart rate monitor to modulate the intensity of exercise, but such set-up has not yet proofed as the best site for pregnant women.

## Conclusions and interpretation

Over a decade, healthcare professionals have mainly focused on overweight/obese women’s exercise during pregnancy related to GDM. However, there is a considerable proportion of GDM women having a normal pre-pregnancy BMI. Also, the majority group in the whole pregnancy population is those with normal pre-pregnancy BMI. From a cost-effectiveness perspective, we need to be more concerned about those women with a normal pre-pregnancy BMI. Our study shows light-to-moderate exercise for 30–60 min, three times a week, during pregnancy is safe and worthy of promotion in normal-weight women with uncomplicated, single pregnancies. This type of exercise could significantly decrease the occurrence of GDM and gestational weight gain, which is associated with adverse outcomes like gestational hypertension, preeclampsia. Besides, exercise during pregnancy is not associated with a reduction of mean gestational age at delivery or an increase in the odds of cesarean delivery. Therefore, our findings support the RCOG recommendations that women with uncomplicated pregnancies should engage in 30 min of moderate physical activity at least four times per week in all trimesters. Our finding indicates that the physical activity intervention in normal pre-BMI women could be a cost-effective or cost-saving management among the pregnancy population with normal pre-pregnancy BMI. Future studies should include larger cohorts to examine the association between exercise pattern (frequency and intensity) and glucose level, and to identify exercise amount and intensity that are suitable for the pregnancy population.

## Additional files

Additional file 1: **Textbox 1.** Search terms used to identify articles related to exercise and gestational diabetes mellitus. (DOCX 12 kb)

Additional file 2: **Figure S1.** Forest plot for the meta-analysis of the gestational weight gain (kg). (PDF 400 kb)

Additional file3: **Figure S2.** Forest plot for the meta-analysis of the gestational age at birth (days). (PDF 514 kb)

Additional file 4: **Figure S3.** Forest plot for the meta-analysis of the birth weight (g). (PDF 593 kb)

Additional file 5: **Figure S4.** Forest plot for the meta-analysis of the odds of caesarean section. (PDF 438 kb)

Additional file 6: **Table S1.** Summary of findings. (PDF 88 kb)

## Abbreviations

BMI: Body mass index
CI: Confidence interval
CVD: Cardiovascular diseases
GDM: Gestational diabetes mellitus
GWG: Gestational weight gain
IADPSG: The International Association of Diabetes and Pregnancy Study Groups
NDDG: National Diabetes Data Group
OR: Odds risk
PRISMA: Preferred reporting items for systematic reviews and meta-analyses
RCOG: Royal College of Obstetricians and Gynecologists
RCT: Randomized controlled trials
SMD: Standard mean differences
T2DM: Type 2 diabetes mellitus
WHO: World Health Organization
