# Supplementary material for: The effect of exercise during pregnancy on gestational diabetes mellitus in normal-weight women: a systematic review and meta-analysis
Source: BMC Pregnancy Childbirth. 2018 Nov 12;18:440. doi: 10.1186/s12884-018-2068-7 (PMC6233372; doi:10.1186/s12884-018-2068-7)
Supplement: Supplementary file 1 — Textbox 1. Search terms used to identify articles related to exercise and gestational diabetes mellitus. (DOCX 12 kb) [file 12884_2018_2068_MOESM1_ESM.docx]

*Supplemental Textbox 1:* Search terms used to identify articles related to exercise and gestational diabetes mellitus

| 1. activit*  2. fitness  3. exercise*  4.sport*  5. physical activit*  6. physical exercise*  7. 1 OR 2 OR 3 OR 4 OR 5 OR 6  8. pregnancy  9. wom*  10. 8 OR 9  11. diabetes  12. gestational diabetes  13. gestational diabetes mellitus  14. GDM  15. glucose  16. 11 OR 12 OR 13 OR 14 OR 15  17. 7 AND 10 AND 16 |
| --- |
