# Supplementary figures and images for: The effect of exercise during pregnancy on gestational diabetes mellitus in normal-weight women: a systematic review and meta-analysis
Source: BMC Pregnancy Childbirth. 2018 Nov 12;18:440. doi: 10.1186/s12884-018-2068-7 (PMC6233372; doi:10.1186/s12884-018-2068-7)

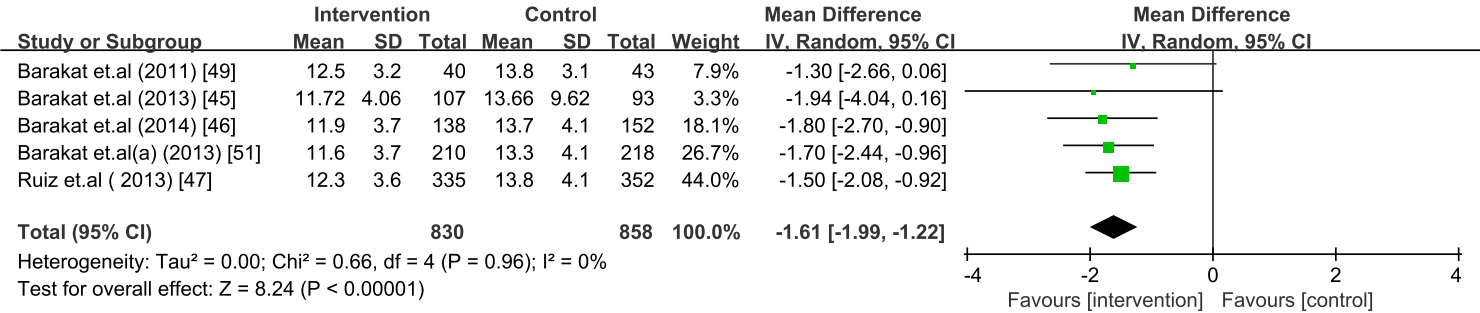

Supplement: Supplementary file 2 — Figure S1. Forest plot for the meta-analysis of the gestational weight gain (kg). (PDF 400 kb) [file 12884_2018_2068_MOESM2_ESM.pdf]

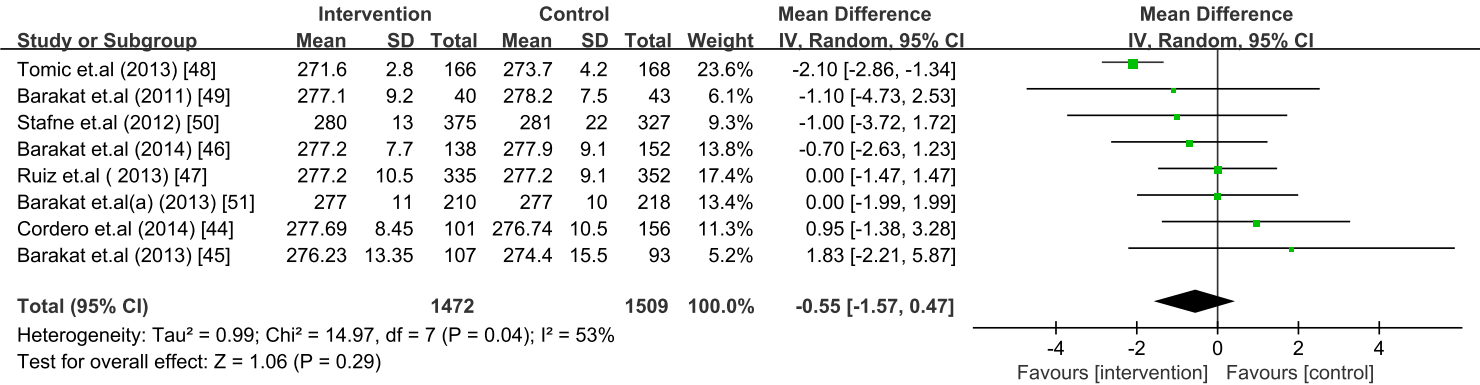

Supplement: Supplementary file 3 — Figure S2. Forest plot for the meta-analysis of the gestational age at birth (days). (PDF 514 kb) [file 12884_2018_2068_MOESM3_ESM.pdf]

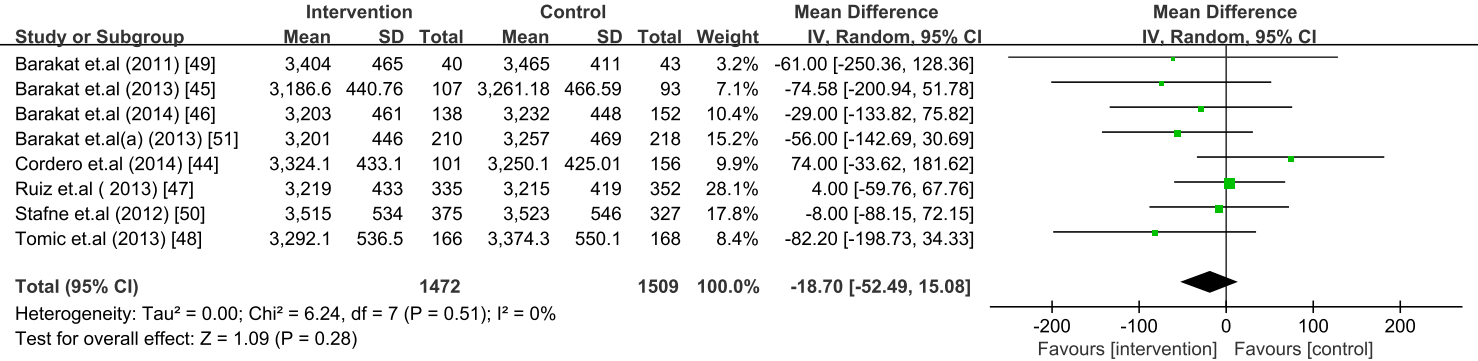

Supplement: Supplementary file 4 — Figure S3. Forest plot for the meta-analysis of the birth weight (g). (PDF 593 kb) [file 12884_2018_2068_MOESM4_ESM.pdf]

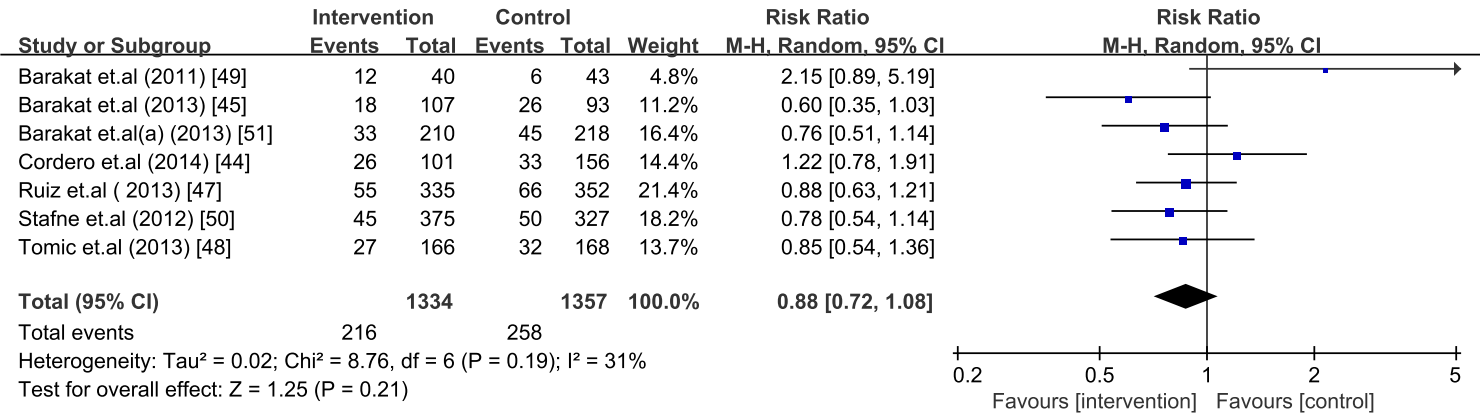

Supplement: Supplementary file 5 — Figure S4. Forest plot for the meta-analysis of the odds of caesarean section. (PDF 438 kb) [file 12884_2018_2068_MOESM5_ESM.pdf]
