# Supplementary material for: The effect of exercise during pregnancy on gestational diabetes mellitus in normal-weight women: a systematic review and meta-analysis
Source: BMC Pregnancy Childbirth. 2018 Nov 12;18:440. doi: 10.1186/s12884-018-2068-7 (PMC6233372; doi:10.1186/s12884-018-2068-7)
Supplement: Supplementary file 6 — Table S1. Summary of findings. (PDF 88 kb) [file 12884_2018_2068_MOESM6_ESM.pdf]

# Summary of findings tables

## 1 The effect of exercise during pregnancy on GDM

| The effect of exercise during pregnancy on GDM | | | | | | |
|---|---|---|---|---|---|---|
| **Patient or population:** patients with GDM<br>**Settings:** Pregnant women<br>**Intervention:** exercise | | | | | | |
| **Outcomes** | **Illustrative comparative risks* (95% CI)** | | **Relative effect (95% CI)** | **No of Participants (studies)** | **Quality of the evidence (GRADE)** | **Comments** |
| | **Assumed risk** | **Corresponding risk** | | | | |
| | **Control** | **Exercise** | | | | |
| **getational diabetes mellitus(1)**<br>follow-up<br>Follow-up: 16-34 weeks | **Study population** | | **RR 0.58**<br>(0.37 to 0.90) | 2981<br>(8 studies) | ⊕⊝⊝⊝ **very low**[1,2,3,4] | |
| | **97 per 1000** | **56 per 1000** (36 to 87) | | | | |
| | **Moderate** | | | | | |
| | **74 per 1000** | **43 per 1000** (27 to 67) | | | | |
| **gestational diabetes mellitus(2)**<br>follow-up<br>Follow-up: 16-34 weeks | **Study population** | | **RR 0.60**<br>(0.36 to 0.98) | 2981<br>(8 studies) | ⊕⊝⊝⊝ **very low**[1,3,4,5] | |
| | **77 per 1000** | **46 per 1000** (28 to 75) | | | | |
| | **Moderate** | | | | | |
| | **73 per 1000** | **43 per 1000** (26 to 71) | | | | |
| **gestational weight gain**<br>Follow-up<br>Follow-up: 16-34 weeks | | The mean gestational weight gain in the intervention groups was<br>-1.61 **lower**<br>(-2.28 lower to 0.54 higher) | | 1690<br>(5 studies) | ⊕⊝⊝⊝ **very low**[1,2,3,4,5] | |
| **gestational age at birth**<br>Follow-up<br>Follow-up: 16-34 weeks | | The mean gestational age at birth in the intervention groups was<br>-0.55 **higher**<br>(-1.57 lower to 0.47 higher) | | 2981<br>(8 studies) | ⊕⊝⊝⊝ **very low**[1,2,3,4,5] | |
| **Birth weight**<br>Follow-up<br>Follow-up: 16-34 weeks | | The mean birth weight in the intervention groups was<br>-18.7 **lower**<br>(-52.49lower to 15.08 higher) | | 2981<br>(7 studies) | ⊕⊝⊝⊝ **very low**[1,2,3,4,5] | |
| **caesarean** | **Study population** | | **RR 0.88** | 2691 | ⊕⊝⊝⊝ | |

| | | | | | | |
|---|---|---|---|---|---|---|
| **section**<br>Follow-up<br>Follow-up: 16-34 weeks | **194 per 1000** | **171 per 1000**<br>(134 to 217) | (0.72 to 1.08) | (7 studies) | **very low**[1,2,3,4,5] | |
| | **Moderate** | | | | | |
| | **204 per 1000** | **180 per 1000**<br>(141 to 228) | | | | |
| *The basis for the **assumed risk** (e.g. the median control group risk across studies) is provided in footnotes. The **corresponding risk** (and its 95% confidence interval) is based on the assumed risk in the comparison group and the **relative effect** of the intervention (and its 95% CI).<br>**CI:** Confidence interval; **RR:** Risk ratio; | | | | | | |
| GRADE Working Group grades of evidence<br>**High quality:** Further research is very unlikely to change our confidence in the estimate of effect.<br>**Moderate quality:** Further research is likely to have an important impact on our confidence in the estimate of effect and may change the estimate.<br>**Low quality:** Further research is very likely to have an important impact on our confidence in the estimate of effect and is likely to change the estimate.<br>**Very low quality:** We are very uncertain about the estimate. | | | | | | |

*Footnotes*

[1] Consistency was downgraded because the heterogeneity across included studies was high.

[2] Precision was downgraded because sample sizes are relatively small in several trials.

[3] Funnel plot indicated publication bias.

[4] Confounding factor, like dietary, may change the effect.

[5] Risk of bias was serious because there existed reporting bias and selection bias.
